# Aerobic capacity and telomere length in human skeletal muscle and leukocytes across the lifespan

**DOI:** 10.18632/aging.102627

**Published:** 2020-01-03

**Authors:** Danielle Hiam, Cassandra Smith, Sarah Voisin, Josh Denham, Xu Yan, Shanie Landen, Macsue Jacques, Javier Alvarez-Romero, Andrew Garnham, Mary N. Woessner, Markus Herrmann, Gustavo Duque, Itamar Levinger, Nir Eynon

**Affiliations:** 1 Institute for Health and Sport (IHES), Victoria University, Melbourne, VIC, Australia; 2 Australian Institute for Musculoskeletal Science (AIMSS), University of Melbourne and Western Health, St Albans, VIC, Australia; 3 RMIT University, School of Health and Biomedical Sciences, Melbourne, VIC, Australia; 4 Clinical Institute of Medical and Chemical Laboratory Diagnostics, Medical University of Graz, Graz, Austria; 5 Department of Medicine-Western Health, Melbourne Medical School, The University of Melbourne, Melbourne, VIC, Australia; 6 Murdoch Children’s Research Institute, Melbourne, VIC, Australia

**Keywords:** exercise, telomere, ageing, aerobic capacity

## Abstract

A reduction in aerobic capacity and the shortening of telomeres are hallmarks of the ageing process. We examined whether a lower aerobic capacity is associated with shorter TL in skeletal muscle and/or leukocytes, across a wide age range of individuals. We also tested whether TL in human skeletal muscle (MTL) correlates with TL in leukocytes (LTL). Eighty-two recreationally active, healthy men from the Gene SMART cohort (31.4±8.2 years; body mass index (BMI)=25.3±3.3kg/m^2^), and 11 community dwelling older men (74.2±7.5years-old; BMI=28.7±2.8kg/m^2^) participated in the study. Leukocytes and skeletal muscle samples were collected at rest. Relative telomere length (T/S ratio) was measured by RT-PCR. Associations between TL, aerobic capacity (VO_2_ peak and peak power) and age were assessed with robust linear models. Older age was associated with shorter LTL (45% variance explained, P<0.001), but not MTL (P= 0.7). Aerobic capacity was not associated with MTL (P=0.5), nor LTL (P=0.3). MTL and LTL were correlated across the lifespan (r_s_=0.26, P=0.03). In healthy individuals, age explain most of the variability of LTL and this appears to be independent of individual aerobic capacity. Individuals with longer LTL also have a longer MTL, suggesting that there might be a shared molecular mechanism regulating telomere length.

## INTRODUCTION

Telomeres are highly conserved repetitive DNA sequences that cap chromosomes [1] and are important in maintaining genetic stability by protecting against genetic recombination, end-to-end fusion of the chromosome and cellular degradation [2, 3]. Somatic cell telomeres shorten with each round of mitotic division until they become too short to divide resulting in cellular senescence [4]. In somatic tissues, telomere length (TL) is established early in life [5, 6], and shortens with older age [7, 8]. Skeletal muscle is a minimally proliferative tissue compared with leukocytes, and therefore, TL in skeletal muscle is in general longer than in leukocytes [6]. The length of telomeres varies considerably between individuals of the same chronological age and is therefore indicative of biological age [8]. The inter-individual variability in TL is thought to be influenced by genetics, epigenetics and environmental factors including oxidative stress, and inflammation [9–12].

TL is associated with some, but not all, ageing and disease-related traits [13, 14]. Literature is inconsistent regarding TL and all-cause mortality with some [7, 15] but not all studies [16, 17] finding that a shorter TL is associated with increased risk of all-cause mortality. Some studies show that a shorter TL is associated with age-related conditions such as cardiovascular disease [18, 19] and diabetes mellitus [9, 20]. While in cancer the literature is conflicted regarding risk of cancer and TL [7, 21–23] with suggestions that perhaps a “U-shaped” risk association may exist between very short and very long telomeres and risk of different types cancer [12, 22, 24, 25]. In two recent papers, there was no association between TL and cognitive function, grip strength, frailty and sarcopenia [13, 26]; suggesting that telomere length may not be a marker of all ageing and disease-related traits.

It is well established that regular physical activity promotes healthy ageing and reduces the risk of developing these chronic diseases [27, 28]. Engagement in exercise is associated with longer telomeres and may slow down TL shortening [29]. Higher aerobic capacity is associated with longer leukocyte TL in endurance trained athletes [30–32] and young (18–32 yrs) [33] and older exercised-trained adults (55–72yrs) when compared to controls [34]. However, the literature regarding TL in skeletal muscle is conflicting, with only few studies reporting a positive association between higher levels of physical activity and longer telomeres in young and older healthy populations [35–37]; while other studies reported no association between skeletal muscle TL and exercise training levels [38, 39]. The variability in findings was highlighted in a systematic review where 54% of studies found no association between physical activity and skeletal muscle TL [40]. Physical activity was assessed using a multitude of subjective measures of physical activity (questionnaires) or motion sensors (accelerometers), which could account for the conflicting results.

To make use of TL as biomarker of ageing and in predicting health outcomes, more research is required to understand the inter-individual variation in TL and its attrition across the human lifespan. Therefore, the aim of this study was to examine whether aerobic capacity (defined using gold-standard measures) is associated with TL in skeletal muscle and/or leukocytes, across a wide age range (18–87 years). We also investigated whether TL in human skeletal muscle is associated with TL in leukocytes.

## RESULTS

The young cohort had a lower BMI, were fitter (higher V̇O2peak and peak power), and harboured longer LTL than the older cohort. However, there was no difference in MTL between the young and old cohort (Table 1).

**Table 1:** Participant characteristics.

**Characteristics**	**Young n=82**	**Older n=11**	**P value**
Age (Years)	31.4 ± 8.3	74.2 ± 7.5	P<0.001
BMI (kg^.^m^-2^)	25.2 ± 3.3	28.7 ± 2.8	P=0.001
VO2peak (mL^.^kg^-1.^min^-1^)	47.3 ± 8.0	20.9 ± 3.4	P<0.001
Peak power (W^.^kg^-1^)	3.7 ± 0.8	1.5 ± 0.3	P<0.001
LTL (AU)	1.24 ± 0.85	0.56 ± 0.27	P<0.001
MTL (AU)	1.06 ± 0.38	0.97 ± 0.25	P=0.45

BMI: Body Mass Index; W: Watts; LTL: Leukocyte Telomere Length; MTL: Skeletal Muscle Telomere Length; AU: arbitrary units. Data is shown as Mean ± SD.

### Fitness parameters and telomere length

**Figure 1:**
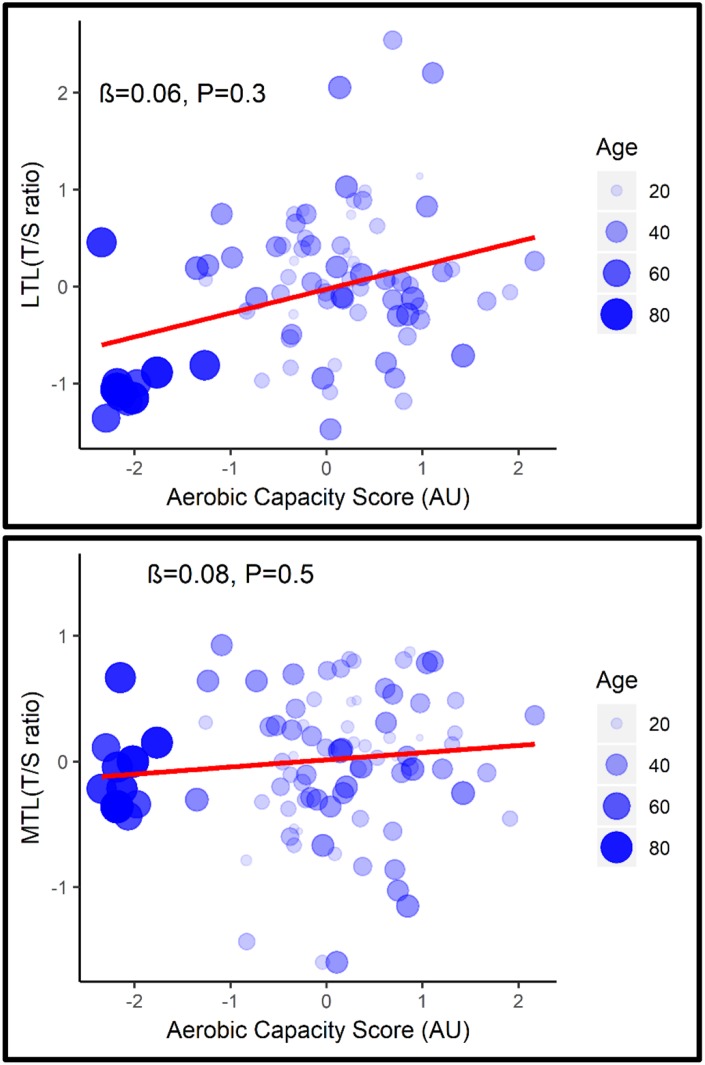
**Association between LTL (top) and MTL (bottom) with aerobic capacity score adjusted for age.**

After adjustment for age, none of the fitness parameters (Supplementary Table 1) or the combined aerobic capacity score were associated with LTL (β= 0.06, P= 0.3; 95% CI= -0.1, 0.2) or MTL (β= 0.08, P= 0.5; 95% CI= -0.1, 0.2) (Figure 1).

**Figure 2:**
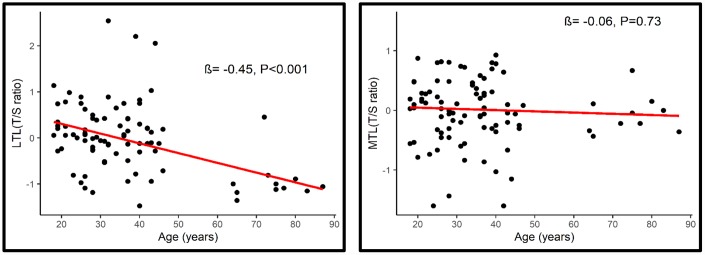
**Association between LTL (Left) and MTL (Right) with age.**

**Figure 3:**
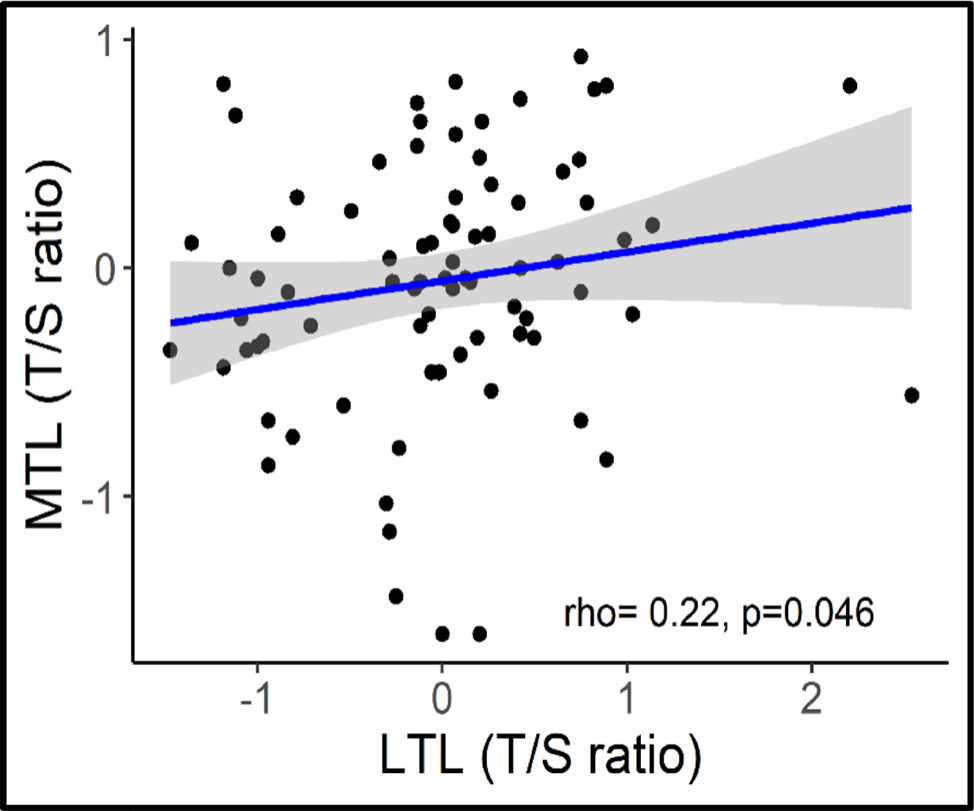
**Correlation between MTL and LTL.**

Overall, age was associated with a shorter LTL (β= -0.45, P<0.001; 95% CI= -0.029, -0.013) but not with MTL (β = -0.06, P=0.73, 95% CI= -0.009, 0.005) (Figure 2). Longer MTL was associated with longer LTL (r_s_=0.22, p=0.046) across the lifespan (Figure 3).

## DISCUSSION

In this study, we confirmed that TL shortens with age in leukocytes of apparently healthy men, and that LTL and MTL are correlated. However, TL was not associated with age in skeletal muscle, and aerobic capacity was not associated with longer telomeres in either leukocytes or skeletal muscle. In addition, a significant correlation was found between LTL and MTL indicating that individuals with a short (or long) telomeres in one tissue also display a short (or long) telomeres in another tissue, consistent with previous reports [6]. Overall, age explains most of the variability of LTL and this appears to be independent of individual aerobic capacity. While MTL appears to be stable (regardless of age and level of fitness) across the lifespan. Further, since it is correlated with LTL, MTL could serve as a reference to indicate shortening in LTL since early-life.

TL in somatic tissues is established early in life [5, 6] and linearly decreases with age after the age of 20 years [7, 8]. The association between TL and age in leukocytes, but not in skeletal muscle, indicates that the TL shortening is variable across different tissues. This is most likely due to the different proliferative capacity and the number cell divisions that take place across the human life-span in each tissue [41, 42]. An inverse correlation between age and LTL was found in our cohort with age accounting for 45% of the variation in TL, which is consistent with literature [8]. It is well known that leukocytes are a highly proliferative tissue and undergo rapid cell division at two different stages of life, during the first years of life and in older age (~60yrs), and hence a strong correlation with age is observed [42, 43]. There is limited data on the association between age and MTL in healthy populations, with most studies having examined the correlation in populations who live with chronic diseases, thus making it difficult to delineate the effect of age and disease status on telomere length [42, 44]. Due to the minimal proliferative capacity of skeletal muscle, it is thought that MTL is maintained throughout adult life and only shortens in response to trauma or disease [45, 46]. Our data also imply a tissue-specific molecular mechanism in skeletal muscle that is maintaining telomere length [45, 47]. In line with this, these data show that age is not associated with MTL in a apparently healthy population, consistent with some [48, 49], but not all reports [6].

We report that a higher LTL is associated with a higher MTL. While LTL undergoes a faster rate of attrition with age than MTL, the inter-individual difference in TL is much larger than the intra-individual difference in TL across tissues [5, 6]. Therefore, an individual with short (or long) telomeres in leukocytes will display corresponding short (or long) telomeres in skeletal muscle. This finding extends knowledge from previous studies which have reported a correlation in foetuses and children [5] and in adults aged between 19–77 years [6]. Overall, these results suggest that the impact of age on telomere attrition is more apparent in highly proliferative tissue than in minimally proliferative tissue.

While the benefits of physical activity on ageing and age-related conditions are well known [27, 28], the benefits on cellular regeneration and senescence are less clear. Our data are in line with a growing body of literature indicating that higher aerobic capacity is not associated with telomere length after adjusting for age [40, 50]. Specifically, we showed that age is the major contributing factor to variability in LTL. We hypothesise that the lack of association may be specific to the population used in this study, i.e. apparently healthy men, with relatively high levels of fitness, according to a recent paper reporting quantile reference values for peak oxygen uptake (VO2peak) [51]. It is possible that TL is maintained at this level of fitness [34, 52]. The literature around TL and self-reported physical activity supports this with those with high levels of habitual physical activity having longer LTL, compared with individuals with low and moderate habitual physical activity [53]. Therefore, perhaps the lack of association of aerobic capacity with TL may due to the high level of fitness exhibited in this cohort and the lack of representation of individuals with a low level of fitness.

A limitation of this study was its cross-sectional nature, and while it would be optimal to conduct a longitudinal study in the same individuals, this was not feasible. We acknowledge that there is a relatively low sample size in our older-adults group (n=11). This relatively small sample size is due to the difficulty to recruit older adults for an invasive study with a muscle biopsy. Telomere length is highly variable between individuals and is impacted by multiple genetic, epigenetic and environmental factors [10, 11, 54, 55]. While we cannot fully account for all these factors, a strength of this study is that our cohort is very homogeneous with regards to confounding factors that can impact TL (i.e. all participants were non-smokers, recreationally active men). Further, we used comprehensive and valid measures of aerobic capacity and created a robust aerobic score to best reflect the aerobic capacity of a person at the physiological level.

## CONCLUSIONS

In apparently healthy individuals, age explains most of the variability of LTL and this appears to be independent of individual aerobic capacity. Individuals with longer LTL also have a longer MTL, suggesting that there might be a shared molecular mechanism regulating telomere length. Whether specific exercise interventions with either high or low intensity components can alter telomere maintenance pathways and regulate telomere length would be interesting to explore in future studies.

## MATERIALS AND METHODS

### Participants

This was a cross-sectional study involving two cohorts of men: the Gene SMART (Skeletal Muscle Adaptive Response to Training) cohort of 82 men aged 18–45 years, and the Wellderly cohort of 11 men aged 64–87 years. Participants’ characteristics are presented in Table 1. All participants were asked to abstain from food, caffeine and alcohol for at least 8 hours prior to blood and muscle collection.

### The gene SMART, young cohort

82 recreationally active (V̇O2peak: 47.3±8.0 mL.kg^-1^. min^-1^) men aged 18–45 years participated in the study following a written informed consent. Volunteers were excluded if they had a chronic disease, were taking medications, warfarin or vitamin K supplementation, or medications that affect metabolism, insulin secretion/sensitivity. Further, participants with known musculoskeletal or other conditions that prevent daily activity were excluded from the study. The full methods of the Gene SMART study are described elsewhere [56]. This study was approved by the Human Ethics Research Committee at Victoria University (HRE13-223).

### The wellderly, older cohort

11 men aged 64–87 years were included in this study. The men were recruited as part of a larger study examining the effect of acute exercise in older-adults. This study has been approved by Melbourne Health (MH) Human Research Ethics Committee and is registered with the Australia New Zealand Clinical Trials Registry ACTRN12618001756213. Volunteers were excluded if they had any fractures in the previous 3 months, or had undergone a new osteoporotic treatment within 3 months or had begun taking anti-resorptive medications within 3 months, have diagnosed diabetes mellitus or were taking hypoglycaemic medications, any haematological, myelodysplastic or myeloproliferative disorder, any bone malignancy, taking warfarin of vitamin K supplementation or restriction, a body mass index > 40 kg/m^2^, or engaged in resistance exercise regime more than 2 sessions per week.

### Skeletal muscle and blood collection

In both cohorts, muscle biopsies were collected from the *vastus lateralis* muscle after an overnight fast by an experienced medical doctor. Following injection of a local anaesthetic (1% Lidocaine), incisions were made, and the biopsy needle inserted. Muscle samples were collected with manual suction applied [56]. Following collection, the samples (50–200 mg) were immediately blotted on filter paper to remove excess blood and placed immediately in liquid nitrogen before being stored at −80 °C until analysis.

Venous blood samples were collected by venepuncture or cannulation just before the muscle biopsy. Five mL of venous blood were collected in EDTA blood collection tubes (Becton, Dickinson and Company, USA), inverted 6–10 times, centrifuged at 3500 rpm for 10 min at 4°C. The plasma was collected and placed in Eppendorf tubes for other analysis. The residual blood sample (which includes the leukocytes) was frozen at -20°C overnight before being stored at −80 °C until DNA extraction.

### Aerobic capacity

### Young cohort

Aerobic capacity was assessed by a graded exercise test (GXT). Briefly, the test consisted of 4-minute exercise stages, separated by 30 second rest periods until voluntary exhaustion [56].

### Older cohort

Peak oxygen consumption (V̇O_2Peak_) was assessed on a cycle ergometer with the initial intensity beginning at 10–30 Watts and increasing by 10–30W×min-1 according to participant capacity. The test was terminated according to participants’ self-reported fatigue perception reaching a pre-determined level (Rate of Perceived Exertion=17) [57] or clinical signs or symptoms. Blood pressure was monitored at baseline, regular intervals (each stage) and post exercise using a manual sphygmomanometer, and heart rate was monitored via the 12-lead ECG.

### Telomere assays

TL was measured in skeletal muscle and leukocytes collected at baseline (in a rested state). Skeletal muscle previously stored at –80°C was lysed with the RLT buffer Plus buffer (Qiagen) and beta-mercaptoethanol using the TissueLyser II (Qiagen, Australia). DNA was extracted using the AllPrep DNA/RNA Mini Kit following the manufacturer guidelines (Qiagen, Australia). DNA yield and purity were assessed using the spectrophotometer (NanoDrop One, Thermofisher) before being stored at –20°C prior to the telomere assays. Genomic DNA was extracted from buffy-coat leukocytes from BD Vacutainer EDTA tubes using the MagSep Blood gDNA kit (0030 451.00, Eppendorf, Hamburg, Germany) [56]. DNA yield and purity were assessed using the spectrophotometer (NanoDrop One, Thermofisher) before being stored at –20°C prior to the telomere assays. The DNA 260/280 and 260/230 OD values were acceptable in the young (1.86 ± 0.07 and 1.35 ± 0.27, respectively) and older (1.93 ± 0.02 and 1.77 ± 0.40, respectively) cohorts.

The telomere (T) to single copy gene (S) ratio (T/S ratio) was calculated to provide telomere length in arbitrary units. Ten microliter reactions comprised of SensiFAST Lo-ROX (20×), 300nM of forward and reverse primers (telomere) or 300nM of forward and 500nM of reverse primers (36B4), H2O and 12.5 ng of DNA template were run in triplicate with no-template negative controls on a single 384-well plate in a thermo-cycler (QuantStudio 12K Flex System, ThermoFisher Scientific, Australia) and repeated twice. The primer sets and thermo-cycling conditions are described elsewhere [30]. The T/S ratios were calculated and expressed relative to the individual with the median T/S ratio using the 2–ΔΔCT method. In skeletal muscle the inter-coefficients of variation for triplicates for the telomere and 36B4 gene were acceptable (mean ± SD: 1.43 ± 0.53 and 1.91 ± 0.57, respectively). In leukocytes inter-coefficients of variation for triplicates for the telomere and 36B4 gene were also acceptable (mean ± SD: 3.61 ± 0.99 and 2.49 ± 0.96, respectively). In skeletal muscle the intra-coefficients of variation for triplicates for the telomere and 36B4 gene were acceptable (mean ± SD: 1.23 ± 0.71 and 1.3 ± 0.74, respectively). In leukocytes the inter-coefficients of variation for triplicates for the telomere and 36B4 gene were also acceptable (mean ± SD: 2.3 ± 1.3 and 1.9 ± 1.2, respectively).

### Statistical analysis

All data were analysed using R studio version 1.1.463. Mann-Whitney tests were used to compare characteristics between the young and older cohorts. The distributions of LTL and MTL were verified for normality visually using histograms and were log-transformed to meet normality assumptions. To examine the additive effect of fitness measures on telomere length, we calculated a fitness score by combining peak power and V̇O2peak into a single value. First, we calculated the z-score for each fitness measure, and then we averaged those z-scores across all fitness measures to calculate the final aerobic capacity score. Robust linear models, using the *rlm* function of the *MASS* package in R [58], were then performed with log-transformed telomere length as the dependent variable, and both age and fitness parameters (peak power, V̇O2peak, or aerobic capacity score) as the independent variables. A likelihood ratio test was used to compare the full model containing both age and fitness parameters (peak power, V̇O2peak, or aerobic capacity score), with a null model containing only age, as implemented in the *lrtest* function of the *lmtest* package in R [59]. Spearman correlations were used to test for an association between MTL and LTL. P values from the statistical analyses were adjusted for multiple testing using the false discovery rate (FDR) [60], and q-values<0.05 were deemed significant.

## Supplementary Material

Supplementary Table 1
