# Supplementary material for: Aerobic capacity and telomere length in human skeletal muscle and leukocytes across the lifespan
Source: Aging (Albany NY). 2020 Jan 3;12(1):359–69. doi: 10.18632/aging.102627 (PMC6977669; doi:10.18632/aging.102627)
Supplement: Supplementary Table 1 [file aging-12-102627-s001..pdf]

SUPPLEMENTARY TABLE

Supplementary Table 1. Association between fitness parameters and MTL or LTL adjusted for age.

	Model adjusted for age		
	VO_{2peak} ($mL.kg^{-1}.min^{-1}$)	Peak Power ($W.kg^{-1}$)	Aerobic Capacity Score (AU)
**MTL**	β= 0.07<br>P= 0.59<br>Q= 0.8	β= 0.13<br>P= 0.4<br>Q= 0.8	β= 0.08<br>P= 0.5<br>Q= 0.8
**LTL**	β= 0.034<br>P= 0.5<br>Q= 0.5	β= 0.07<br>P= 0.3<br>Q= 0.45	β= 0.06<br>P= 0.3<br>Q= 0.45

AU: Arbitrary Units; LTL: Leukocyte Telomere Length; MTL: Skeletal Muscle Telomere Length. P-values were adjusted for multiple testing using the false discovery rate (FDR). Effect size corresponds to the regression coefficient in the linear models and is interpreted as the change in MTL or LTL (log-transformed) adjusted for age per unit change in fitness parameter.
